# Pyoderma Gangrenosum: An Ulcer With Unorthodox Treatment

**DOI:** 10.7759/cureus.19324

**Published:** 2021-11-07

**Authors:** Tridip Dutta Baruah, Rubik Ray, Manju R

**Affiliations:** 1 Department of General Surgery, All India Institute of Medical Sciences, Raipur, IND

**Keywords:** pathergy, immunomodulator, non-healing ulcer, dermatosis, pyoderma gangrenosum

## Abstract

Pyoderma gangrenosum (PG) is a rare, reactive, non-infectious inflammatory dermatosis. It typically presents with extensive cutaneous ulcerations.

A 20-year-old lady presented with a painful, progressive, non-healing ulcer with purulent discharge on the right upper thigh. Debridement of the ulcer was done, and pus was sent for culture and sensitivity. Despite regular wound care, the ulcer was progressing in size with persistent pain. The ulcer exhibited the phenomenon of pathergy. The pus was sterile on examination, and the histopathology showed extensive neutrophilic infiltration. A history of similar non-healing ulcers in a family member pointed toward the diagnosis of this rare condition.

Treatment of pyoderma gangrenosum started in conjunction with the dermatology department. After appropriate wound care with systemic steroids and immunomodulators, the ulcer healed by secondary intention. PG is a diagnosis of exclusion. A high level of suspicion of an uncommonly presenting ulcer would lead to early diagnosis and appropriate treatment. Early diagnosis and treatment with corticosteroids and immunosuppressants can heal the lesion early by minimizing pathergy.

## Introduction

Pyoderma gangrenosum (PG) is a rare, reactive, non-infectious, inflammatory dermatosis. It typically presents with extensive cutaneous ulcerations. In 1908, the French dermatologist Louis Brocq first reported a series of patients with characteristic features of pyoderma gangrenosum. Later in 1930, Brunsting et al. first introduced the term pyoderma gangrenosum [1].

We present a case of pyoderma gangrenosum of the lower limb, in which surgical intervention led to an inadvertent worsening of the clinical situation and exhibited the phenomenon of pathergy. Pyoderma gangrenosum is a diagnosis of exclusion. A high level of suspicion of an uncommonly presenting ulcer would lead to early diagnosis and appropriate treatment. Early diagnosis and treatment with corticosteroids and immune-suppressants can heal the lesion early by minimizing pathergy.

The case was previously presented as a poster in CGASICON 2020 (19th Annual Conference of The Association of Surgeons of India - Chhattisgarh Chapter).

## Case presentation

A 20-year-old lady noticed a small, pea-sized, painful ulcer with purulent discharge over the postero-lateral aspect of the right upper thigh with fever for the past 15 days. The ulcer was diagnosed as infective and treated by debridement in a local hospital. However, the ulcer was not biopsied and the symptoms aggravated with progress in size with persistent pain, for which she presented to our OPD.

**Figure 1:**
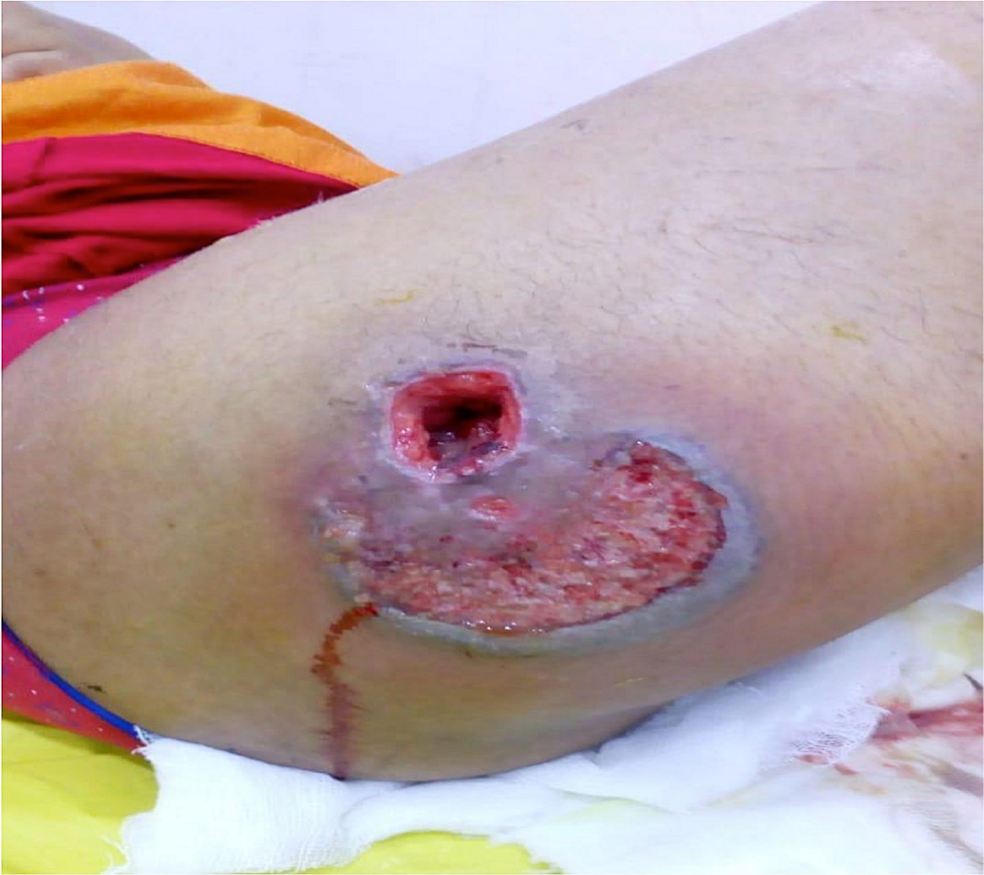
Ulcer at presentation

On examination, the patient was febrile. The local examination revealed a burrowed ulcer of 2*2cm and a nearby ulcer of 4*3cm with a cribriform appearance over the back of the right thigh. Margins of the ulcer were edematous, ragged, tender, and a seropurulent discharge was present. There was no history of trauma prior to the development of the lesion, no joint pain, no blood in stools, or loose stools. There were no similar lesions elsewhere on the body (Figure 1).

Initial laboratory investigation showed a total leucocyte count of 15400 (polymorphs-81%) cells/mm^3^. A diagnosis of spreading ulcer due to infectious etiology was made, and a decision for debridement was taken. All unhealthy tissue was excised and sent for histopathological examination and bacterial culture and sensitivity.

**Figure 2:**
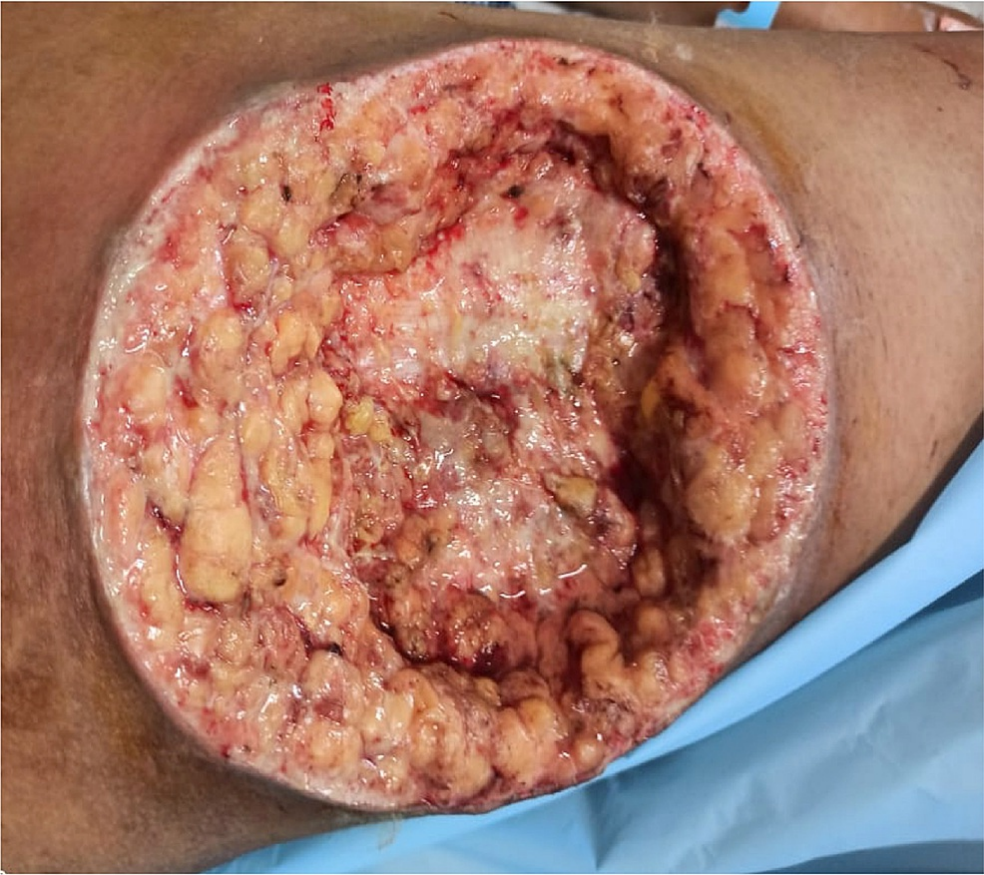
Wound five days after debridement

Postoperatively, the patient was put on broad-spectrum antibiotics. However, the condition of the wound worsened despite regular wound care. The ulcer size progressed to 20*15 cm within a week with an inflamed and tender margin with a violaceous hue. The edges and the ulcer floor were filled with slough and pale granulation tissue (Figure 2).

Bacterial culture and sensitivity revealed no growth, and the histopathology report revealed epidermal necrosis with extensive neutrophilic infiltration. After taking an extensive family history, it was found that a family member of the patient had a similar ulcer that had to be treated with steroids and took months to heal. The diagnosis of pyoderma gangrenosum was made, and treatment started in conjunction with the department of dermatology.

**Figure 3:**
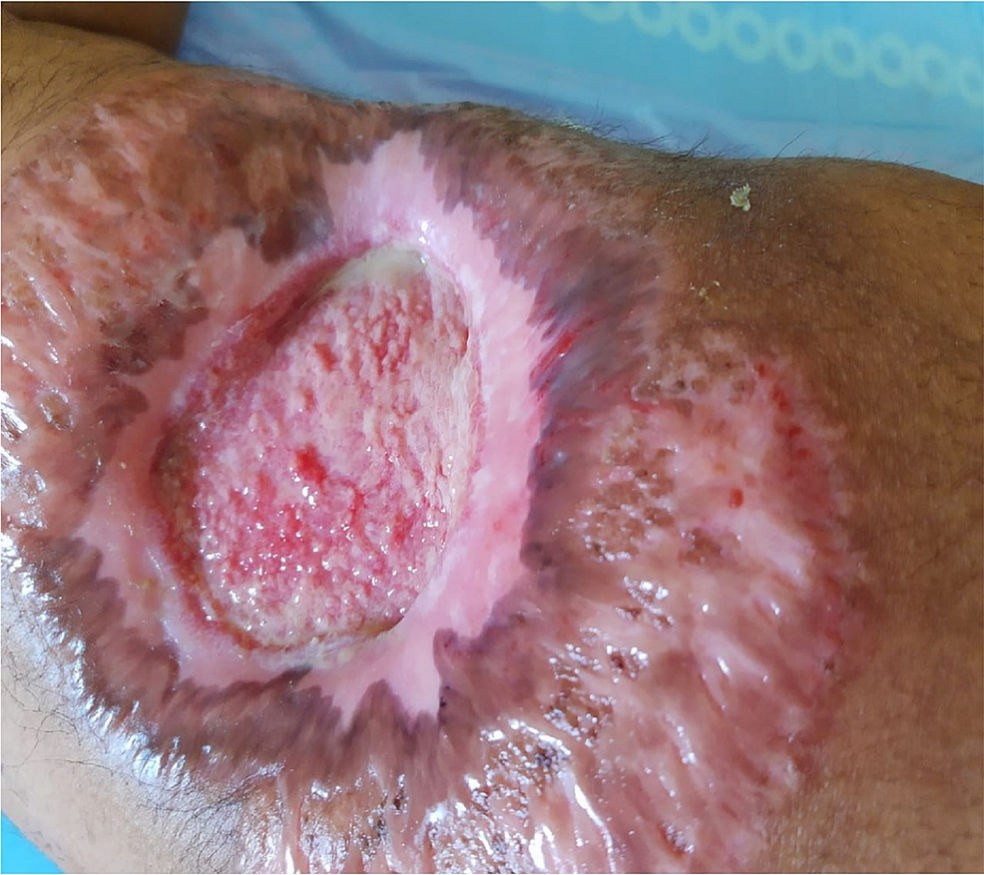
Wound healing by scarring after two and half months of treatment

**Figure 4:**
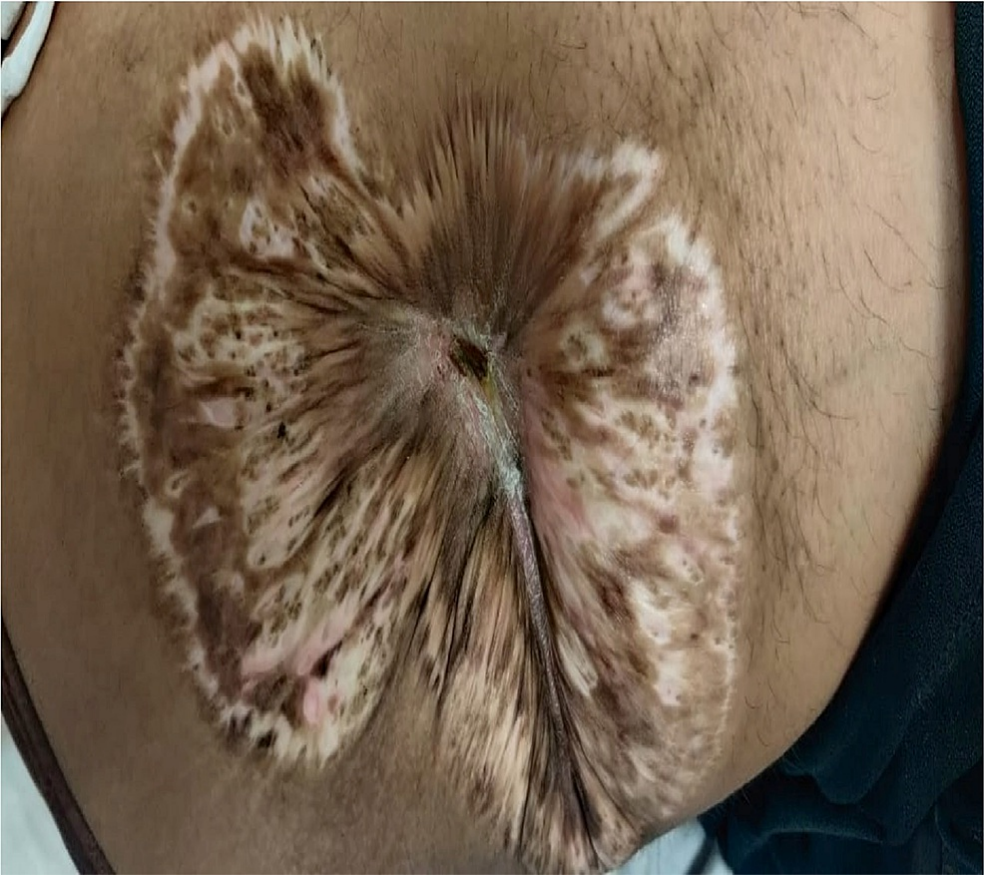
Wound completely healed by scarring after four months of treatment

For wound care, negative pressure wound therapy was applied, followed by weekly platelet-rich plasma dressings. Intravenous dexamethasone (8 mg OD), and oral cyclosporine (100 mg BD) were started initially. Later, methylprednisolone pulse therapy was considered. Cyclosporine was tapered in view of systemic side effects, and the patient had to be started on mycophenolate mofetil (1 gm BD). After four months of intensive treatment with regular wound care, the ulcer slowly healed by scarring (Figures 3-4).

## Discussion

Pyoderma gangrenosum is a reactive non-infectious inflammatory dermatosis. It falls under the spectrum of neutrophilic dermatoses. The worldwide incidence reported is around two to three cases per 100,000 per year. The peak incidence occurs between the ages of 20-50 years with a possible slight female preponderance [2].

There are several subtypes. ‘Classical PG’ is the most common form and is seen in approximately 85% of cases. This presents as an extremely painful erythematous lesion that rapidly progresses to a blistered or necrotic ulcer. There is often a ragged undermined edge with a violaceous/erythematous border. The lower legs are most frequently affected although PG can present at any body site. Other subtypes include bullous, vegetative, pustular, peristomal, and superficial granulomatous variants [3]. It is frequently associated with inflammatory bowel diseases (IBD), inflammatory arthropathies, and hematologic malignancies [4]. The differential diagnosis includes all other causes of cutaneous ulceration, including infections, vasculitis/vasculopathy, and neoplastic disorders.

Early diagnosis of PG is difficult, as there are no definitive laboratory or histopathological criteria for diagnosing PG. PG is a diagnosis of exclusion. The workup starts with a cutaneous biopsy that preferentially includes the border of the ulcer and the adjacent skin. Histology is essential to exclude vasculitis and malignancy. Special stains and tissue cultures may also be used to rule out infection [1]. Massive neutrophilic infiltration in the absence of vasculitis and granuloma formation is typical of PG [2].

There are no established guidelines for the treatment of PG, however, first-line treatment is aimed at optimizing the care of wounds. Surgical treatment of active PG ulcers was controversially discussed due to the pathergy phenomenon, which presents with new PG lesions or the rapid progression of existing ulcers after tissue traumatization or surgery. Without surgical intervention and systemic immunosuppression, ulcers take months to years for healing.

Potent topical corticosteroids and tacrolimus ointment can be applied to the ulcer surface [5]. Skin graft coverage with hyperbaric oxygen therapy, skin grafting with negative pressure wound therapy under the coverage of immunosuppressant is found to be a better option for wound outcomes [6-7]. The best surgical approach reported is split-thickness skin graft (STSG) secured by negative pressure wound therapy (NPWT), as this leads to higher graft uptake [7]. During the regression of PG, the border of the lesion's collapses, erythema fades and granulation tissue appears on the ulcer. After healing, atrophic, cribriform scars persist [8].

Systemic corticosteroids form the mainstay of immunosuppressive treatment. Prednisolone (0.5-1 mg/kg/day) is usually preferred. Ciclosporin can be used either alone or in combination with corticosteroids as a steroid-sparing agent in cases where prolonged treatment is required [9]. Dapsone can be preferred as an anti-inflammatory steroid-sparing agent over cyclosporine, especially in elderly patients, as it does not affect renal function or blood pressure and for its well-known additional antimicrobial properties [10]. Other systemic treatments tried with varying success include colchicine, sulphasalazine, minocycline, apremilast, and thalidomide. Pulsed intravenous methylprednisolone can be useful in initiating a rapid response given together with immunosuppressive drugs such as methotrexate, mycophenolate mofetil, cyclophosphamide, azathioprine, and high-dose intravenous immunoglobulin. The highest rate of adverse events per therapeutic attempt has been described to occur with cyclosporine (40%), followed by cyclophosphamide (38%) and azathioprine (32%), in a large study on 52 patients by Herberger et al. [11].

Due to a better understanding of pathogenesis and more and more clinical reports, a shift toward the usage of biologic treatments is seen and a shift from biologicals targeting TNF-alpha (infliximab) to IL-12/23 (ustekinumab) or IL-23 antibodies is preferred, as the latter shows good clinical responses with fewer side effects [11]. However such modalities are still to be available for routine use.

## Conclusions

Pyoderma gangrenosum is a disease with poorly understood pathology exhibiting pathergy and recurrence. It is wiser for all clinicians to consider a diagnosis of pyoderma gangrenosum among the differential diagnosis when dealing with painful cutaneous ulcerative lesions, especially in patients with a positive family history or underlying systemic illness. Frequently, the ulcer is misdiagnosed as infective and treated by local therapies like debridement or incision and drainage, which further exacerbate the lesion. Usually, the patient is treated with myriad therapies along with systemic antibiotics before arriving at the 'unlikely' diagnosis. 

Pyoderma gangrenosum needs to be identified earliest for better outcomes. Steroid and immunosuppressive therapy are useful in early healing with minimal complications.
